# Presenting signs and symptoms of artificial urinary sphincter cuff erosion

**DOI:** 10.1590/S1677-5538.IBJU.2022.0089

**Published:** 2022-03-18

**Authors:** Linley Diao, Samantha W. Nealon, Gianpaolo P. Carpinito, Shervin Badkhshan, Avery R. Wolfe, Benjamin M. Dropkin, Sarah C. Sanders, Steven J. Hudak, Allen F. Morey

**Affiliations:** 1 Department of Urology University of Texas Southwestern Medical Center Dallas Texas USA Department of Urology, University of Texas Southwestern Medical Center, Dallas, Texas, USA

**Keywords:** Urinary Incontinence, Urinary Sphincter, Artificial, Male

## Abstract

**Purpose:**

To characterize the most common presentation and clinical risk factors for artificial urinary sphincter (AUS) cuff erosion to distinguish the relative frequency of symptoms that should trigger further evaluation in these patients.

**Materials and Methods:**

We retrospectively reviewed our tertiary center database to identify men who presented with AUS cuff erosion between 2007 – 2020. A similar cohort of men who underwent AUS placement without erosion were randomly selected from the same database for symptom comparison. Risk factors for cuff erosion – pelvic radiation, androgen deprivation therapy (ADT), high-grade prostate cancer (Gleason score ≥ 8) – were recorded for each patient. Presenting signs and symptoms of cuff erosion were grouped into three categories: obstructive symptoms, worsening incontinence, and localized scrotal inflammation (SI).

**Results:**

Of 893 men who underwent AUS placement during the study interval, 61 (6.8%) sustained cuff erosion. Most erosion patients (40/61, 66%) presented with scrotal inflammatory changes including tenderness, erythema, and swelling. Fewer men reported obstructive symptoms (26/61, 43%) and worsening incontinence (21/61, 34%). Men with SI or obstructive symptoms presented significantly earlier than those with worsening incontinence (SI 14 ± 18 vs. obstructive symptoms 15 ± 16 vs. incontinence 37 ± 48 months after AUS insertion, p<0.01). Relative to the non-erosion control group (n=61), men who suffered erosion had a higher prevalence of pelvic radiation (71 vs. 49%, p=0.02).

**Conclusion:**

AUS cuff erosion most commonly presents as SI symptoms. Obstructive voiding symptoms and worsening incontinence are also common. Any of these symptoms should prompt further investigation of cuff erosion.

## INTRODUCTION

Despite its wide acceptance and high treatment success, the artificial urinary sphincter (AUS) remains prone to complications requiring replacement or removal of the device in an estimated one third of patients ( 1 - 5 ). Urethral cuff erosion remains one of the more common and most devastating long-term complications. Although cuff design updates have decreased erosion rates since the device’s inception, recent long-term observational series continue to suggest that approximately 8% of patients undergoing AUS placement will eventually develop a cuff erosion ( 1 - 5 ).

To date, most literature on AUS cuff erosion focuses on its risk factors. History of prior pelvic radiation has been associated with both a shorter time to and higher likelihood of cuff erosion ( 5 - 12 ). Other implicated risk factors include hypertension ( 12 ), diabetes ( 13 ), cardiovascular disease ( 12 , 13 ), low testosterone ( 14 ), urethral catheter ( 15 ), penile prosthesis placement ( 16 ), prior urethral surgery ( 9 , 17 , 18 ), and prior cuff erosion ( 7 , 9 , 10 , 19 , 20 ).

Despite abundant literature on medical conditions linked with AUS cuff erosion, less information exists addressing the specific presenting signs and symptoms of this troublesome condition. Signs and symptoms which have been attributed to cuff erosion include hematuria, dysuria, and recurrent SUI ( 21 - 23 ). We predicted that physical exam findings of scrotal inflammation predict AUS cuff erosion. Herein, we review the presenting signs and symptoms of AUS cuff erosion cases from our tertiary center in an effort to promote timely identification by clinicians, thereby facilitating intervention prior to the development of additional local or systemic complications.

## MATERIALS AND METHODS

We retrospectively reviewed our large tertiary center database, identifying men who presented with AUS cuff erosion between 2007 and 2020 (IRB: STU-2020-1187). The primary endpoint was to identify presenting signs and symptoms of cuff erosion. A secondary objective was to gauge clinical risk factors for cuff erosion – for this analysis, a comparison control group of the same size was randomly selected from our AUS database of men without AUS cuff erosion using a number generator tool.

Established risk factors for cuff erosion – pelvic radiation, androgen deprivation therapy (ADT), and high-grade prostate cancer (Gleason score ≥ 8) – were recorded for each patient. Presenting signs and symptoms of cuff erosion were identified by chart review of patient notes in the electronic medical record system. History and exam findings were grouped into three categories: obstructive symptoms, worsening incontinence, and localized scrotal inflammation (SI) around the AUS pump i.e. “pump-itis”. We also evaluated signs and symptoms at follow up of our non-eroded control cohort.

Demographic data were collected and compared between symptom groups. Multivariable logistic regression was employed to assess for any association between presenting symptoms and time to cuff erosion. All statistical analyses were performed in SPSS (Armonk, NY: IBM Corp.) with p<0.05 considered statistically significant.

## RESULTS

**Figure 1:**
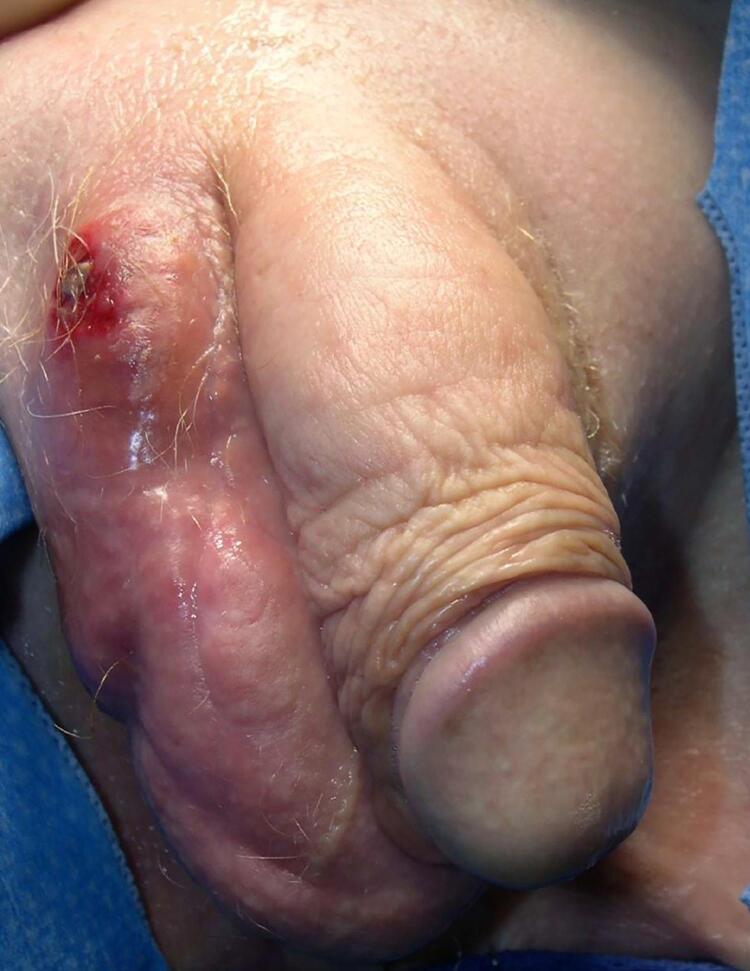
Photo representation of a patient with Scrotal Inflammation, i.e, “Pump-itis”

**Figure 2:**
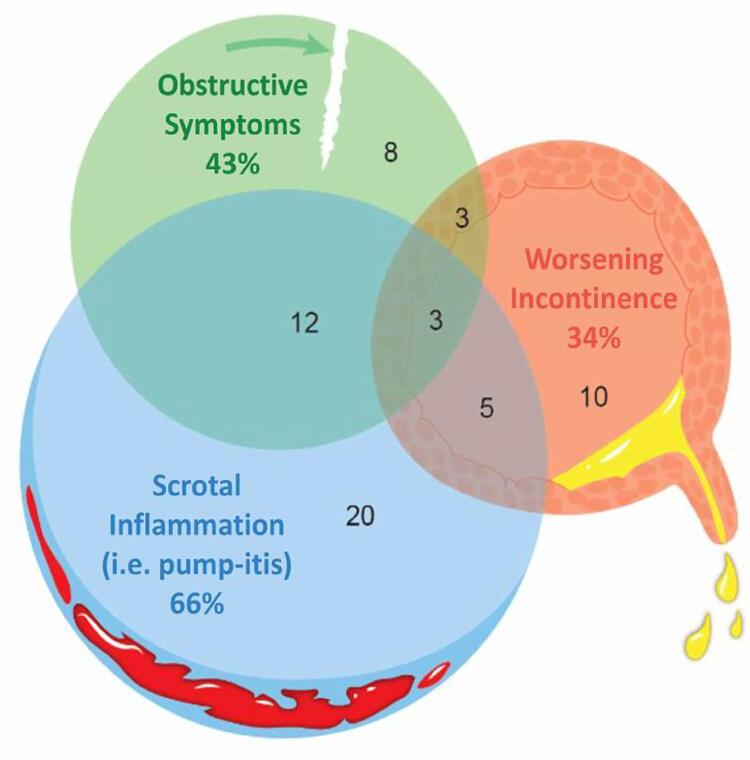
Presenting Signs and Symptoms of Artificial Urinary Sphincter Cuff Erosion

Among 893 men who underwent AUS placement during the period examined, 61 (6.8%) sustained cuff erosion. The average age at time of AUS removal was 74.8±7.2 years old. No patients in either group had tandem cuffs. Most erosion patients (40/61, 66%) presented with SI changes including tenderness, erythema, and swelling ( Figure-1 ). Fewer men reported obstructive symptoms (26/61, 43%) and worsening incontinence (21/61, 34%). Three AUS cuff erosions presented with all three groups of symptoms - SI, obstructive symptoms, and worsening incontinence (3/61, 5%). Roughly one-third presented with two out of three symptom groups – SI and obstructive symptoms (12/61, 20%), compared to the less common combinations of SI and worsening incontinence (5/61, 8%) and obstructive symptoms and worsening incontinence (3/61, 5%, Figure-2 ). The average length of time from AUS placement to cuff erosion was 22.2 months ± 33.7. Men with SI or obstructive symptoms presented significantly earlier than those with worsening incontinence (SI 14 ± 18 vs. obstructive symptoms 15 ± 16 vs. incontinence 37 ± 48 months after AUS insertion, p<0.01).

In the non-eroded control group, office notes from the most recent follow up visit (written by either the primary investigator or reconstruction fellows) indicated that the majority (55/61, 90.2%) presented without complaints, were pleased with their device, and satisfied with dryness, with either complete continence or with very mild incontinence requiring one safety pad per day. Of the 6 with any complaint, 4/6 had worsening incontinence of more than two pads per day, one had urge urinary incontinence, and one had chronic penile pain unrelated to his AUS. All (61/61, 100%) presented with a normal exam, characterized by documentation of “no inflammation, swelling, redness, or tenderness” on examination, with normal pump positioning and no evidence of pressure-regulating balloon herniation.

Prevalence of comorbidities was evaluated in the erosion cohort - hypertension (53, 86.9%), diabetes mellitus (20, 32.8%), coronary artery disease (33, 54.1%), smokers (43, 70.5%), ( Table-1 ). Of note, 43 men (70.5%) had a history of radiation for the treatment of prostate cancer. Other prior treatments included prostatectomy (47, 77%), prior AUS placement (31, 50.8%), urethroplasty (15, 24.6%), transurethral resection of prostate (5, 8.2%), prolonged catheterization with AUS in place (6, 9.8%). Of these 61 cases, nine had a prior AUS cuff erosion (15%). Cuffs were replaced transcorporally in 8/9 (89%) patients.

**Table 1:** Patient demographics and treatment history.

	Overall n=61	Scrotal Inflammation n=40	Obstructive Symptoms n=26	Worsening Incontinence n=21	P-value
**Patient Demographics**					
Age at AUS Removal mean, (st dev)	74.86 (7.21)	74.28 (7.35)	75.69 (6.41)	75.30 (6.96)	0.702
BMI at AUS Removal mean, (st dev)	28.58 (5.12)	28.85 (4.90)	29.09 (5.31)	27.29 (4.04)	0.395
Months to Erosion mean, (st dev)	22.19 (33.75)	14.19 (18.8)	15.38 (16.61)	37.38 (48.94)	0.009
HTN	53 (86.9%)	36 (90.0%)	22 (84.65%)	16 (76.2%)	0.355
Diabetes	20 (32.8%)	15 (37.5%)	9 (34.6%)	7 (33.3%)	0.558
CAD	30 (49.2%)	24 (60.0%)	14 (53.8%)	7 (33.3%)	0.061
Smoking	43 (70.5%)	28 (70.0%)	16 (61.5%)	16 (90.4%)	0.548
**Treatment History**					
Radiation	43 (70.5%)	30 (75.0%)	19 (73.1%)	10 (47.6%)	0.088
Prostatectomy	49 (80.3%)	33 (82.5%)	19 (73.1%)	16 (76.2%)	0.504
TURP	4 (6.5%)	2 (5.0%)	1 (3.8%)	2 (9.5%)	0.681
Prior Urethroplasty	12 (19.6%)	8 (20.0%)	4 (15.4%)	4 (19.1%)	0.891
Prior AUS Placement	28 (45.9%)	18 (45.0%)	10 (38.5%)	12 (57.1%)	0.436

**AUS** = artificial urinary sphincter; **St dev** = standard deviation; **BMI** = body mass index; **HTN** = hypertension; **CAD** = coronary artery disease; **TURP** = transurethral resection of prostate

Relative to the non-erosion control group (n=61), men who eroded had higher rates of pelvic radiation (71 vs. 49%, p=0.02, see Table-2 ). They also had higher rates of hypertension (87 vs. 64%, p=0.003), coronary artery disease (54 vs. 12 %, p<0.00001), and smoking history (71 vs. 51%, p=0.03). Rates of treatment with ADT (41 vs. 38 %, p=0.77), high-grade prostate cancer (39 vs. 39 %, p=0.98), and comorbid diabetes (33 vs. 20%, p=0.09) were similar. There were no statistically significant relationships found between patient demographics, comorbidities, or treatment history and presenting symptoms of AUS cuff erosion ( Table- 1).

**Table 2:** Demographic and Treatment History – Erosion vs Non-Erosion Cohort.

	Erosion (n=61)	Non-Erosion (n=61)	P-Value
Pelvic Radiation	43 (71%)	30 (49%)	**0.02**
Hypertension	53 (87%)	39 (64%)	**0.00**
Coronary artery disease	33 (54%)	7 (12%)	**0.00**
Smoking	43 (71%)	31 (51%)	**0.03**
Androgen deprivation therapy	17 (41%) n=42	23 (38%)	0.70
High grade prostate cancer	9 (39%) n=23	24 (39%)	0.98
Diabetes	20 (33%)	12 (20%)	0.09

## DISCUSSION

This series highlights the typical clinical presentation of AUS cuff erosion – a devastating scenario for both incontinence patients and their urologists. Men with severe AUS cuff erosion are prone to develop secondary complications including urethral stricture, diverticulum, and fistula (26). These complications often necessitate additional surgeries which can further disrupt any chance for acceptable continence. We believe that earlier recognition facilitates expedient treatment, thereby reducing risk of attendant complications and hastening recovery.

### Presenting signs and symptoms of erosion

Anecdotal reports suggest that late obstructive symptoms and worsening incontinence are potential signs of cuff erosion that should prompt cystoscopy ( 21 , 23 ). The present large case series underscores these concepts but advances the importance of SI symptoms (“pump-itis” - scrotal tenderness, erythema, and swelling around the pump) as the most common early manifestations of AUS cuff erosion. We hypothesize the SI develops due to ongoing urinary seepage from the urethra, passing along the AUS tubing to the pump, where it becomes secondarily inflamed and in many cases, overtly infected.

Notably, more than half of men with erosion who expressed a complaint of obstructive symptoms also complained of SI and vice-versa ( Figure-2 ). Although each of these individual symptoms should prompt suspicion for cuff erosion, their combination especially suggests a high reliability for this serious complication.

### Time to erosion

Men with recurrent SUI were diagnosed with AUS cuff erosion significantly later than men without this symptom. Prior studies report a wide range of time to erosion from 1.9 months to 3 years ( 2 , 4 , 8 ). From our data, it is not possible to determine the underlying reason for later erosion identification in these men. We hypothesize that progressive cuff erosion leads to worsening SUI that only becomes apparent to the patient and/or provider when a certain threshold of bother is reached. In these cases, it is alternatively possible that cuff erosion was present asymptomatically for an extended time while another time-dependent process, such as urethral atrophy or mechanical failure, independently led to incontinence and delayed evaluation ( 1 , 2 , 7 , 24 ).

### Erosion post-radiation and additional risk factors

Our finding of increased risk of cuff erosion in patients with history of pelvic radiation is consistent with prior studies ( 5 - 12 ). Supporting the concept that microvascular and histologic tissue changes after radiation negatively impact tissue integrity ( 25 ). We did not identify differences in cuff erosion rates for those with prior transurethral resection of prostate, urethroplasty, or other medical comorbidities ( Table-1 ). Power remains an issue in confirming any of the above relationships, as only a small fraction of AUS patients had undergone any of the above interventions. For men in the erosion cohort, average testosterone level at time of erosion was 222.0 ng/dL±177 ng/dL (IQR 237.5). As previously described, low testosterone is a known risk factor for AUS cuff erosion ( 14 ). We did not have testosterone levels for the non-erosion cohort as these are not routinely drawn.

### Limitations

We recognize several limitations of our study. Although the retrospective design limits the inference of causal relationships, as a descriptive study, this design was suitable for our primary aims. We suspect that some patients were lost to follow up or followed up with their local urologists as we operate at a large tertiary referral center, thus introducing an attrition bias. We believe that patients with complications are more likely to follow up, leading to selection bias. As a single center study, results may have been impacted by surgeon technique and patient population factors, though these are unlikely to have affected our primary endpoint. There is an intrinsic difficulty in identifying patients with cuff erosion given a lack of established guidance in the literature about presenting symptoms of erosion, but the work-up is almost always symptom-driven.

We did not perform routine cystourethroscopy on the control cohort to rule out subclinical erosion, so it is unclear whether any small cuff erosions remain asymptomatic in our patient population or if any may have been asymptomatic with significant lead time prior to identification. Urinalyses as well as urine and device cultures were not consistently performed on this patient group, so these findings were not included in our study. Only 5 of the erosion patients complained of gross hematuria at presentation, so this symptom was not included as a presentation group. There are several areas for future study direction on this topic. It would be interesting to determine whether the severity of clinical presenting signs and symptoms of erosion correlate with larger degree of urethral cuff erosion and also whether the size of erosion affects final outcomes for patients as it relates to long term urethral patency, complications, repeat infections, and ability to have another AUS inserted at a later date.

## CONCLUSION

AUS cuff erosion most commonly presents with scrotal inflammatory symptoms. Obstructive voiding symptoms and worsening incontinence are also common. Patients with prior pelvic radiation are at a higher risk of AUS cuff erosion. Heightened awareness of these common clinical presentations may aid in prompt identification and subsequent timely treatment of cuff erosions.
